# Supplementary material for: Content-rich biological network constructed by mining PubMed abstracts
Source: BMC Bioinformatics. 2004 Oct 8;5:147. doi: 10.1186/1471-2105-5-147 (PMC528731; doi:10.1186/1471-2105-5-147)
Supplement: Additional File 2 — The original results of the above study (non-essential files are deleted to keep the file size under the limit set by BMC bioinformatics). [file 1471-2105-5-147-S2.bz2 › chilibotAdditionalFile2/dip20/628ID11089964E11015/html/SRP9_SRP14.html]

 


 **SRP9** and **SRP14** 
  
Found 21 abstracts in PubMed, retrieved 20.  
 

 What does Google say? 
 PDF only 
| .edu only 

---

**Interactive relationship** (e.g. stimulation, inhibition, etc)

**Neutral relationship**- The proteins  **SRP9**  and  **SRP14**  first heterodimerize and then initially bind to the Alu RNA 5 domain.  Ref: 11350037 RNA, 2001
- This elongation arrest activity requires the  **SRP9**  14 subunit of the particle and interactions of the C terminus of  **SRP14** .  Ref: 10921896 EMBO J, 2000
- **SRP9**  and  **SRP14**  proteins form a heterodimer that binds to the Alu domain of SRP RNA which is responsible for translation arrest.  Ref: 9233785 EMBO J, 1997
- Two polypeptides of the murine signal recognition particle SRP,  **SRP9**  and  **SRP14** , bind exclusively as a heterodimer to SRP RNA and their presence is required for elongation arrest activity of the particle.  Ref: 9214658 RNA, 1997
- **SRP9**  and  **SRP14**  form a heterodimer which specifically binds to SRP RNA.  Ref: 7518078 Nucleic Acids Res, 1994
- The  **SRP9**  protein binds only as dimer with  **SRP14**  protein to the Alu domain of 7SL RNA to form a subdomain that, in SRP, is functional in translation arrest.  Ref: 9605471 Neurosci Lett, 1998
- We generated a truncated  **SRP14**  protein,  **SRP14**  20C, which forms, together with  **SRP9** , a stable complex with SRP RNA.  Ref: 9115358 Nucleic Acids Res, 1997

**Non-interactive relationship** (e.g. studied together, co-existance, homology, etc.)

- In  **SRP9** , acidic amino acid residues in the N terminal alpha helix and the adjacent loop and, in  **SRP14** , a flexible internal loop region are critical for RNA binding.  Ref: 9214658 RNA, 1997
- This release includes 11 new SRP RNA sequences total of 129, two protein  **SRP9**  sequences total of seven, two protein  **SRP14**  sequences total of 10, two protein SRP19 sequences total of 1 0 new SRP54 ffh sequences total of 66, two protein SRP68 sequences total of seven and two protein SRP72 sequences total of nine.  Ref: 11125080 Nucleic Acids Res, 2001
- This release includes 82 SRP RNAs including 22 bacterial and 9 archaeal homologs and a total of 20 protein sequences representing  **SRP9** ,  **SRP14** , SRP19, SRP54, SRP68, and SRP72.  Ref: 7524020 Nucleic Acids Res, 1994
- This release adds three SRP RNA sequences totaling 96 SRP RNA sequences and 11 SRP protein sequences a total of 39 protein sequences from  **SRP9** ,  **SRP14** , SRP19, SRP21, SRP54, SRP68 or SRP72.  Ref: 9016514 Nucleic Acids Res, 1997
- The 9 kD  **SRP9**  and the 14 kD  **SRP14**  subunits of SRP are required to confer elongation arrest activity to the particle.  Ref: 7518078 Nucleic Acids Res, 1994
- these RNPs accumulate after adenovirus infection, while levels of  **SRP9** ,  **SRP14** , SRP54 and 7SL SRP RNA are unaffected.  Ref: 8932367 Nucleic Acids Res, 1996
- The current release included 93 RNAs and 29 proteins representing  **SRP9** ,  **SRP14** , SRP19, SRP21, SRP54, SRP68 and SRP72.  Ref: 8594607 Nucleic Acids Res, 1996
- Recombinant SRPphi14 9 is a fusion protein containing on a single polypeptide chain the sequences of both the  **SRP14**  and  **SRP9**  proteins that are part of the Alu domain of the signal recognition particle SRP.  Ref: 9409618 RNA, 1997
- **SRP9**  and  **SRP14**  are found to be structurally homologous, containing the same alpha beta beta beta alpha fold.  Ref: 9233785 EMBO J, 1997
- In addition, the human  **SRP9**  14 binds with higher affinity than mouse  **SRP9**  14 to all RNAs analyzed and this difference is not explained by the additional C terminal domain present in the anthropoid  **SRP14** .  Ref: 9016560 Nucleic Acids Res, 1997
- We have constructed cDNAs that encode single polypeptide chains comprising  **SRP9**  and  **SRP14**  sequences in the two possible permutations linked by a 17 amino acid peptide.  Ref: 7518078 Nucleic Acids Res, 1994
- The Alu domain of the mammalian signal recognition particle SRP comprises the heterodimer of proteins  **SRP9**  and  **SRP14**  bound to the 5 and 3 terminal sequences of SRP RNA.  Ref: 11089964 Nature, 2000
- html adds four SRP RNA sequences a total of 99 SRP RNA sequences, 23 SRP protein sequences a total of 63 protein sequences from  **SRP9** ,  **SRP14** , SRP19, SRP21, SRP54, SRP68 or SRP72, and, for the first time, sequences of the alpha subunit of the eukaryotic SRP receptor and its homologous bacterial proteins a total of 21 sequences.  Ref: 9399828 Nucleic Acids Res, 1998
- Although  **SRP9**  proteins are the same size in both rodent and primate cells,  **SRP14**  subunits are generally larger in primate cells.  Ref: 7542942 Mol Biol Cell, 1995
